# Downregulation of Nodal inhibits metastatic progression in retinoblastoma

**DOI:** 10.1186/s40478-019-0785-4

**Published:** 2019-08-26

**Authors:** Laura Asnaghi, David T. White, Lynn Yoon, Antoinette Price, Grace Y. Lee, Arpan Sahoo, Jeff S. Mumm, Charles G. Eberhart

**Affiliations:** 1 Department of Pathology, Johns Hopkins University, School of Medicine, Smith Building, 400 N. Broadway Avenue, Room 4029, Baltimore, MD 21287 USA; 2 Department of Ophthalmology, Johns Hopkins University, School of Medicine, Baltimore, MD USA; 3 Department of Oncology, Johns Hopkins University, School of Medicine, Baltimore, MD USA

**Keywords:** Nodal, Invasion, Proliferation, Retinoblastoma

## Abstract

Retinoblastoma is the most common intraocular malignancy in children. We previously found that the ACVR1C/SMAD2 pathway is significantly upregulated in invasive retinoblastoma samples from patients. Here we studied the role of an ACVR1C ligand, Nodal, in regulating growth and metastatic dissemination in retinoblastoma. Inhibition of Nodal using multiple short hairpin (shRNAs) in WERI Rb1 and Y79 retinoblastoma cell cultures reduced growth by more than 90%, as determined by CCK-8 growth assay. Proliferation was also significantly inhibited, as found by Ki67 assay. These effects were paralleled by inhibition in the phosphorylation of the downstream effector SMAD2, as well as induction of apoptosis, as we observed more than three-fold increase in the percentage of cells positive for cleaved-caspase-3 or expressing cleaved-PARP1. Importantly, we found that downregulation of Nodal potently suppressed invasion in vitro, by 50 to 80%, as determined by transwell invasion assay (*p* = 0.02). Using an orthotopic model of retinoblastoma in zebrafish, we found 34% reduction in the ability of the cells to disseminate outside the eye, when Nodal was knocked down by shRNA (*p* = 0.0003). These data suggest that Nodal plays an important role in promoting growth, proliferation and invasion in retinoblastoma, and can be considered a new therapeutic target for both primary tumor growth and metastatic progression.

## Introduction

Retinoblastoma is a tumor of the retina and the most frequent intraocular cancer in children, accounting for 3% of all pediatric malignancies [31]. It affects about 250–300 children per year in the United States and is responsible for about 3000–4000 deaths annually worldwide [11, 17]. Early diagnosis and aggressive treatment strategies have made near-complete cure rates possible in developed countries, where the survival rate has reached almost 100% [9]. However, current treatments can have negative impacts on vision. In addition, retinoblastoma still remains a potentially blinding, debilitating, and fatal tumor in developing countries, where early diagnosis, intensive chemotherapy and long-term follow-up are not as readily available, leading to a drop in the worldwide survival rate to 50% [6, 7, 37]. With early detection, in most cases the primary tumor can be successfully treated by systemic or local chemotherapy [9]. However, these therapies can be associated with sight-threatening complications and increased risk for secondary malignancies, particularly in the cases with germline *RB1* mutations [28, 29]. Moreover, metastases to the central nervous system or in distant organs, including bone and bone marrow, are resistant to chemotherapy and thus represent a serious life-threatening complication. Therefore, novel therapeutic options are actively being pursued for advanced retinoblastoma, as well as for the primary tumor, in order to find new therapeutic targets to block metastatic spread and decrease the risks associated with systemic or local chemotherapy, utilized to treat the primary tumor.

In our previous study we demonstrated that genetic and pharmacological blockade of the Activin A receptor type 1C (ACVR1C), also known as Activin-like kinase receptor 7 (ALK7), strongly inhibited both primary growth and metastatic spread of retinoblastoma cells [3]. Here we focused on the role of the ligands of ACVR1C receptor, which include Nodal, Activin and growth/differentiation factor 3 (GDF3), to determine the origin of this pro-metastatic signaling in retinoblastoma cells. The intrinsic serine/threonine kinase activity of the ACVR1C receptor is induced by interaction with these ligands, which results in phosphorylation of the SMAD2/3/4 complex, promoting its nuclear translocation and activation of gene transcription [23]. ACVR1C ligands control many physiological processes, such as proliferation, differentiation, and wound healing. In particular, Nodal plays fundamental roles during embryonic development, where it is crucial for left-right axis specification of visceral organs [14, 22] and for regulating germ cell versus somatic cell fate decisions in early mouse development [30]. Nodal is also important for the maintenance of human embryonic stem cells [13, 25] and has a pro-tumorigenic effect in several tumor types. Moreover, Nodal signaling is involved in retinal development, inducing the formation of retinal progenitor cells from mouse embryonic stem cells [5]. Nodal also regulates differentiation of WERI Rb1 cells into retinal neurons [21]. Here we focused on dissecting the role of ACVR1C ligands, as they represent an intriguing and promising point of therapeutic intervention to suppress the activity of the receptor and downstream signaling, which we have shown is crucial in promoting metastatic progression in retinoblastoma [3]. We focused specifically on Nodal, as we found previously that it was more highly expressed in multiple retinoblastoma cell lines, as compared to the other ligands of the ACVR1C receptor [3].

## Materials and methods

### Cell culture and reagents

WERI-Rb1 [24] and Y79 [27] human retinoblastoma cells lines were purchased from American Type Culture Collection (ATCC, Manassas, VA) and cultured in RPMI-1640 medium supplemented with 50 IU/ml penicillin, 50 μg/ml streptomycin, 1% L-glutamine and 10% heat-inactivated fetal bovine serum (FBS), at 37 °C in a humidified 5% CO_2_ atmosphere. All cell lines were tested periodically for mycoplasma contamination and STR profiling. pLKO.1 vectors containing short hairpin RNA (shRNA) targeting Nodal (sequences are described in Table 1) or scrambled shRNA (used as a control) were purchased from Thermo Fisher (Waltham, MA). Preparation of the lentiviral particles containing Nodal or scrambled shRNAs was carried out as previously described, using HEK293T cells as a packaging system [2]. Selection of the cells expressing Nodal or scrambled shRNAs was performed using Puromycin (1 μg/mL). Knock down of Nodal expression was evaluated by Western blot, after cells were selected with Puromycin for at least 10 days.

**Table 1:** Sequences of Nodal shRNAs

	CLONE ID	Vector	Target gene	Sequence
A	TRCN0000058699	pLKO.1	Nodal (NM_018055.5)	CCGGGCGGTTTCAGATGGACCTATTCTCGAGAATAGGTCCATCTGAAACCGCTTTTTG
B	TRCN0000058701	pLKO.1	Nodal (NM_018055.5)	CCGGTGCCACCAATGTGCTCCTTATCTCGAGATAAGGAGCACATTGGTGGCATTTTTG
C	TRCN0000058702	pLKO.1	Nodal (NM_018055.5)	CCGGCATAAAGACATGATCGTGGAACTCGAGTTCCACGATCATGTCTTTATGTTTTTG
D	TRCN0000429895	pLKO.1	Nodal (NM_018055.5)	CCGGGCTCACTTGCCATTGAGATTTCTCGAGAAATCTCAATGGCAAGTGAGCTTTTTTG
E	TRCN0000424693	pLKO.1	Nodal (NM_018055.5)	CCGGGCATGCTGTATGTGGATAATGCTCGAGCATTATCCACATACAGCATGCTTTTTTG

### Clinical specimens and immunohistochemical staining

Expression of Nodal was determined by immunohistochemistry (IHC) on 5-μm-thick sections of 12 formalin-fixed paraffin-embedded surgical specimens derived from enucleated eyes resected from patients diagnosed with retinoblastoma at the Wilmer Eye Institute (Johns Hopkins University), with local institutional review board approval. Previously described immunostaining techniques were utilized [2]. In brief, slides were incubated overnight at room temperature with anti-Nodal mouse monoclonal antibody (Sigma-Aldrich, #SAB1404135, St. Louis, MO), diluted 1:500 in 2% normal goat serum/0.1% Triton X100/TBS. Secondary antibody was purchased from Vector Laboratories, Burlingame, CA (anti-mouse: #PK6102) and diluted 1:200 in 2% normal goat serum/0.1% Triton X100/TBS. Nodal immunostaining was scored in 12 samples by a board certified pathologist (C.G. Eberhart), and the intensity of the staining was scored as 0: no expression; 1+: weak expression; 2+: moderate expression; 3+: strong expression in over 50% of the cells. Murine intraocular xenografts of Y79 cells were used as a positive control for the specificity of the primary antibody. Y79 xenograft sections not treated with primary anti-Nodal antibody were used as a negative control, along with internal negative controls such as blood vessels.

### Western blotting

Protein levels of total and phospho-SMAD2, ZEB1, Snail, and cleaved poly (ADP-ribose) polymerase (PARP1) were evaluated by Western blot in retinoblastoma cells, with β-Actin used as a loading control. Proteins were extracted using TNE lysis buffer, as previously described [2], and equal amounts loaded into 4–12% SDS-polyacrylamide gels for electrophoresis-based separation (Invitrogen, Carlsbad, CA). Proteins were then transferred on a nitrocellulose membrane (Invitrogen) and incubated for 1 h in blocking solution containing 5% dried milk in TBS with 0.1% Tween 20 (TBS-T). Membranes were incubated with the primary antibody overnight in blocking solution at 4 °C. The following primary antibodies were used: Nodal (in mouse, 1:800, Sigma-Aldrich, #SAB1404135), total and phospho-SMAD2 (in rabbit, 1:1000, Cell Signaling Technology, #5339, #18338, Danvers, MA), ZEB1 (in rabbit, 1:2000, Sigma-Aldrich, #HPA027524, St. Louis, MO), Snail (in mouse, 1:1000, Cell Signaling Technology, #3895, Danvers, MA), cleaved poly (ADP-ribose) polymerase 1 (PARP1) at Asp^214^ (in rabbit, 1:1000, Cell Signaling Technology, #5625), β-Actin (in mouse, 1:500, Santa Cruz Biotechnology, #sc-47,778, Dallas, TX). Secondary antibodies bound to peroxidase and raised in mouse or in rabbit (1:3000, Cell Signaling Technology, #7074, #7076) were used to visualize the protein bands. Enhanced chemiluminescence (ECL) was used as the detection reagent (PerkinElmer, Waltham, MA).

### Growth and proliferation assays

Cell Counting-Kit 8 (CCK-8, Sigma-Aldrich), containing WST-8 reagent [2-(2-methoxy-4-nitrophenyl)-3-(4-nitrophenyl)-5-(2,4-disulfophenyl)-2H-tetrazolium, monosodium salt], was utilized to measure cell growth, as previously described [2]. Cell proliferation was determined by Ki67 immunoassay, using Muse® Cell Analyzer (Millipore, Billerica, MA), following the manufacturer’s protocol for non-adherent cells.

### Apoptosis

Induction of apoptosis in retinoblastoma cells upon suppression of Nodal was determined by immunofluorescence assay, using cleaved caspase-3 antibody (in rabbit, 1:400, Cell Signaling Technology, #9661), as previously described [34]. Images were taken using DS-Fi3 Nikon camera and processed with NIS-Elements D software (Melville, NY).

### Transwell invasion assay

The ability of the cells to invade Matrigel was determined by transwell invasion assay, as previously described [3]. Equal amount of cells were plated in each insert, in 10% FBS medium. An FBS gradient (0 to 10%) was established between the medium in the insert (0%) and in the well (10%), to induce chemotaxis of the cells from the insert to the well. After incubation for 72 h, the amount of viable cells that had migrated through the filter of the insert, pre-coated with Matrigel (diluted 1:10), were counted by trypan blue exclusion dye. Only the unstained/viable cells were counted, excluding the possibility that reduction in cell invasion could be attributable to apoptosis.

### In vivo analysis in zebrafish

Zebrafish background strain was “AB” (Zebrafish International Resource Center, ZIRC). Zebrafish were maintained using standard temperature and light cycle conditions (28.5 °C, 14 h of light/10 h of dark). All experimental procedures were approved by the Animal Care and Use Committee of Johns Hopkins University. For zebrafish xenotransplantation, a procedure previously described was followed [3]. Approximately 80 Y79 cells, labelled with GFP-MSCV retroviral vector [1], and treated with Nodal shRNA or scrambled shRNA controls, were injected (Dagan PMI-100 microinjector) into the vitreous cavity of each embryo, at 2 days post fertilization (dpf). Larvae were transferred to an incubator and maintained at 28.5 °C overnight. At 1 day post-injection (dpi) larvae were screened for a visible GFP^+^ cell mass at the injection site via stereo fluorescence microscopy (Olympus SZX16, Center Valley, PA). The localization of the GFP expressing retinoblastoma cells was monitored by confocal intravital microscopy (Olympus FV1000) at 1 and 4 dpi, to determine whether reduction in Nodal modified the metastatic potential of the retinoblastoma cells to migrate outside the eye. The extent of retinoblastoma metastasis was determined using IMARIS & Matlab software, as previously described [3, 35]. Images were processed to remove the diffuse green autofluorescence due to the endogenous pigmentation of the zebrafish, which was accounted for during the MBS analysis.

### Statistical analysis

Experiments were carried out in biological triplicate and the data are presented as the mean + standard deviation (SD). Levels of significance were determined by two-sided Student *t*-test, with *p*-values lower than 0.05 considered statistically significant. Statistical calculations were performed using GraphPad Prism7 software (San Diego, CA). For the in vivo analyses, data were processed with a custom R-based package (ggplot2, 59) to generate box plots showing the first quartile (lower box), median (bold line), third quartile (upper box), upper and lower adjacent (whiskers), and raw data (dot plot; large dots denote outlier observations) for each experimental condition. Statistical analyses were done using R3.3.1 and RStudio 0.99.893. Student’s *t* test was used to calculate effect size between paired groups, with effect size, 95% confidence intervals (CI), and *p*-values provided.

## Results

### Expression of Nodal protein in human retinoblastoma specimens

**Fig. 1:**
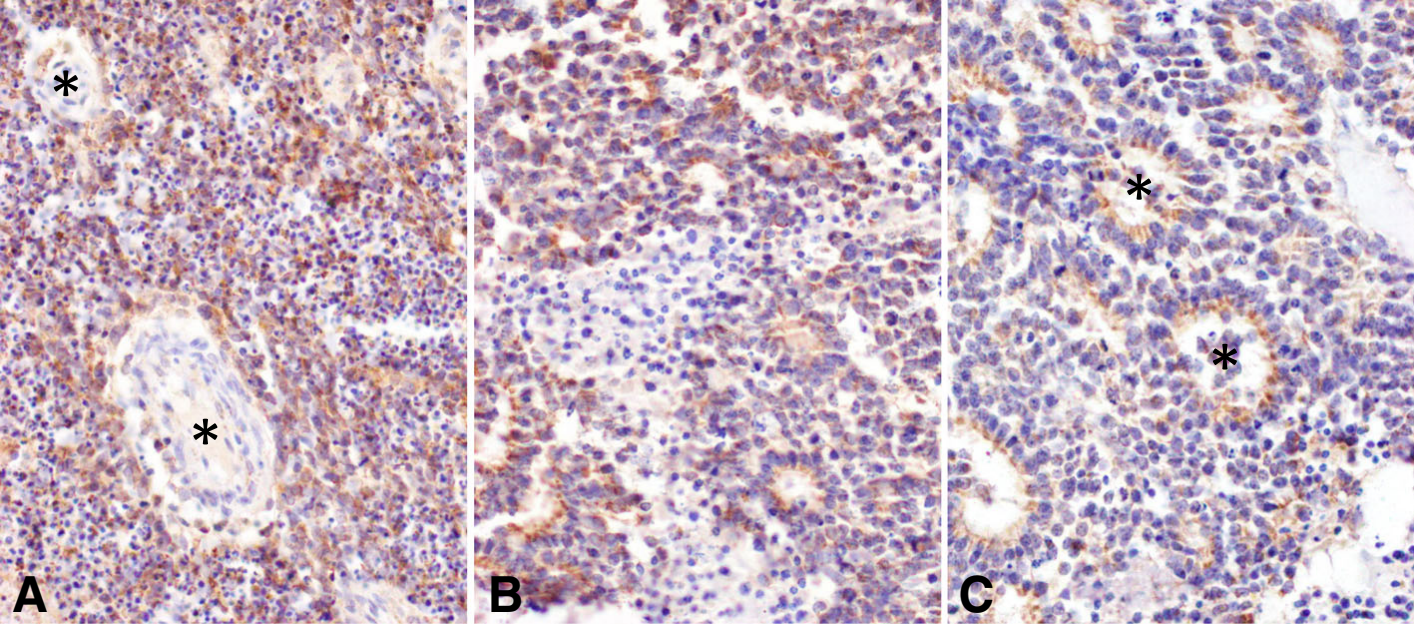
Expression of Nodal protein in retinoblastoma. **a.** Strong, diffuse expression of Nodal in retinoblastoma cells, with two vessels (asterisks) largely negative for the protein on immunohistochemical analysis serving as internal negative controls. **b.** Strong expression of Nodal in the periphery of a retinoblastoma. **c**. In the tumor shown in b, Nodal expression was only moderate in the center of the lesions, and was most prominent in the neoplastic rosettes (asterisks). (Original magnification 200X in **a**, and 400X in **b** and **c**)

We examined the expression of Nodal protein in 12 human retinoblastoma specimens using immunohistochemical analysis. Staining was noted in the cytoplasm and on the surface of tumor cells, while stromal elements such as blood vessels were negative and served as an internal control (Fig. 1a). Interestingly, in 3 cases with prominent Flexner-Wintersteiner rosettes, expression was highest in the luminal cytoplasm of the neoplastic cells forming the rosettes. Overall, expression was strong in 3 cases (25%), moderate in 6 cases (50%), weak in 2 cases (16.7%) and negative in 1 case (8.3%). In 8 of the tumors which expressed Nodal, the staining was only present focally, and it was generally strongest in the peripheral portions of the lesions, although more central regions of tumor were also sometimes positive to a lesser degree (Fig. 1b,c). The final 3 positive cases showed more diffuse staining.

### Downregulation of Nodal inhibits invasion and induces apoptosis in retinoblastoma

**Fig. 2:**
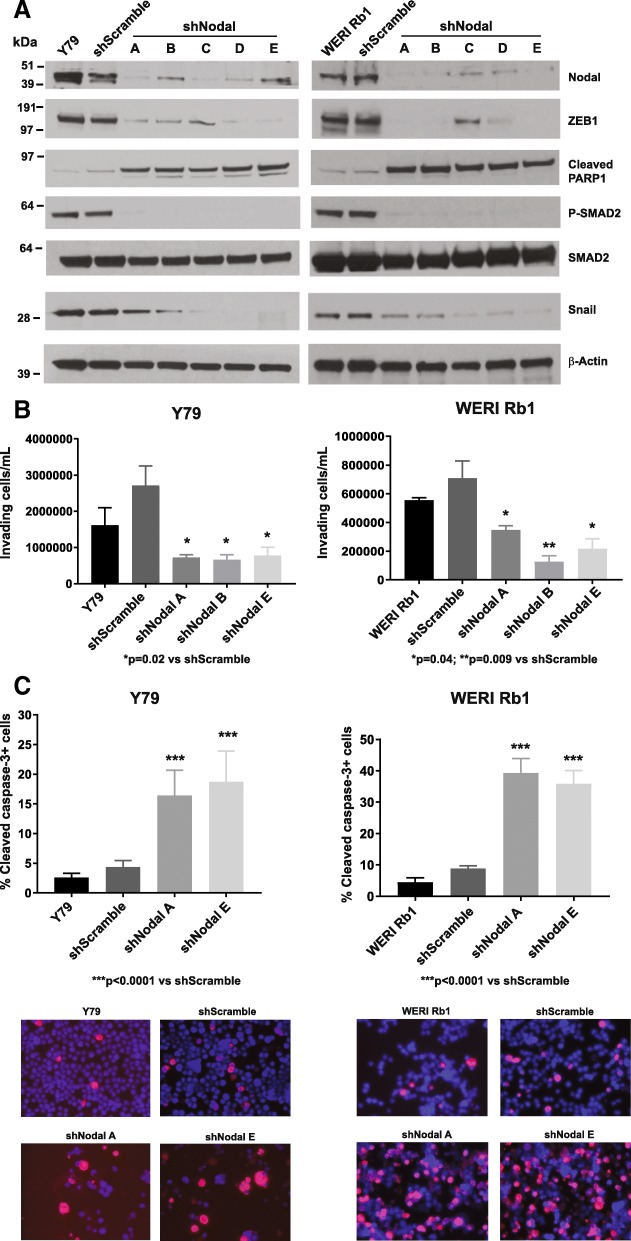
Nodal downregulation inhibits invasion and induces apoptosis in retinoblastoma. **a** Nodal protein levels were dramatically reduced by five different shRNAs, as determined by Western blot, using β-Actin as a loading control. This downregulation was paralleled by a profound reduction in ZEB1 and Snail protein levels, and inhibition of phospho-SMAD2, whereas the protein levels of total SMAD2 did not change. Cleaved-PARP1, a marker of late apoptosis, was also induced by the downregulation of Nodal. **b** The ability of the cells to invade a Matrigel-coated filter was reduced in Y79 cells expressing shRNAs targeting Nodal, compared to scrambled shRNA controls, as determined by transwell invasion assay. **c** Induction of apoptosis upon Nodal downregulation was also confirmed by immunofluorescence assay using an antibody specific for cleaved caspase-3 (red). Nuclei were stained with DAPI (blue). P values were calculated using two-sided Student t-test comparing cells expressing Nodal shRNA and scrambled shRNA. Data are presented as mean + SD. Microphotographs in the lower part of the panels are representative images of the immunofluorescence staining for cleaved caspase-3 (magnification: 40X)

We have previously found high expression of the ligand Nodal at the mRNA and protein levels in multiple retinoblastoma lines, including Y79 and WERI Rb1 [3]. To test the role of Nodal in tumor growth and metastatic potential, expression was inhibited by shRNAs in these lines. Five different target sequences reduced Nodal protein levels by more than 90% as determined by Western blot (Fig. 2a). Downregulation of Nodal with all five shRNAs correlated with a reduction in phospho-SMAD2, indicating pathway suppression, and with a profound decrease in the expression of the epithelial-to-mesenchymal transition (EMT) markers ZEB1 (*zinc finger E-box binding homeobox* 1) and Snail, as found by Western blot (Fig. 2a). In both cell lines, reduction in ZEB1 and Snail protein expression was paralleled by inhibition in the ability of the retinoblastoma cells to invade Matrigel: between 50 to 80% reduction in transwell invasion was observed upon suppression of Nodal (Fig. 2b). Reduction in Nodal expression also induced apoptosis, with increased levels of cleaved PARP1, a marker of late apoptosis, evident by Western blot (Fig. 2a). This finding was consistent with a substantial increase in the percentage of cells positive for cleaved-caspase-3, another marker of late apoptosis, as determined by immunofluorescence assays in Y79 and WERI Rb1 cells (Fig. 2c).

### Nodal suppression inhibits growth and proliferation of retinoblastoma cells

**Fig. 3:**
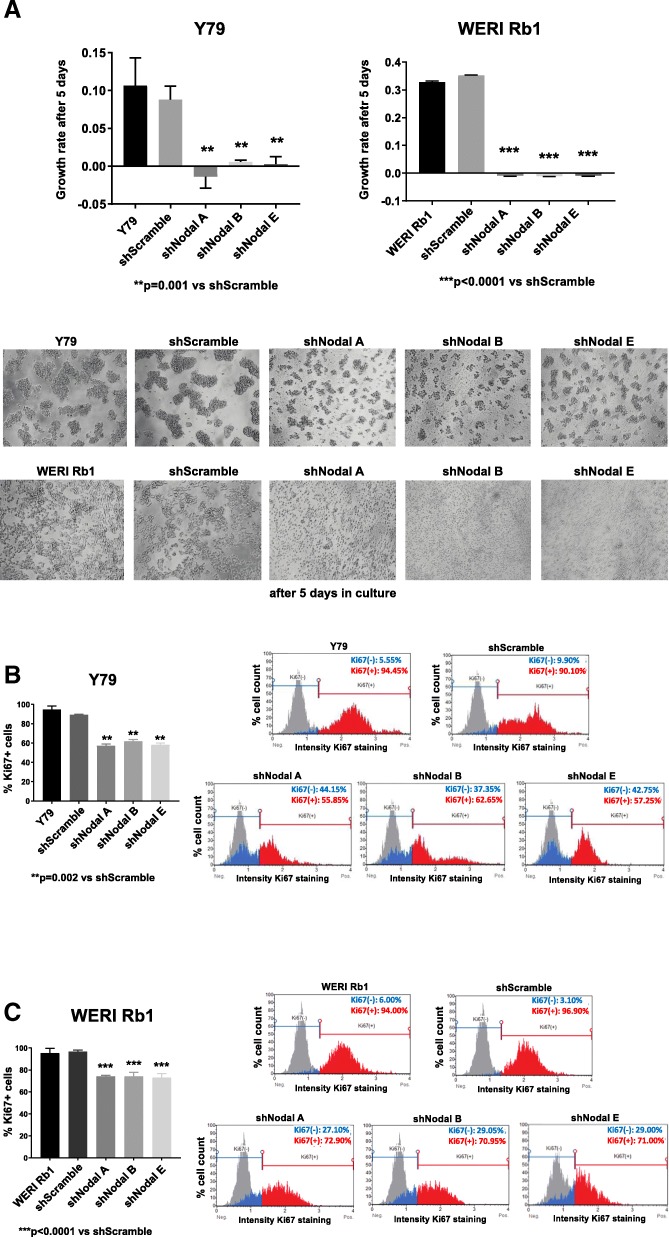
Nodal knock down inhibits growth and proliferation of retinoblastoma cells. **a** Growth was inhibited by more than 90% in Y79 and WERI Rb1 cells expressing Nodal shRNAs, compared to scrambled shRNA controls, as determined by CCK-8 growth assay. Microphotographs in the lower part of the panel are representative images of the cells after 5 days in culture (magnification: 10X). Nodal downregulation produced smaller aggregates in Y79 and single cell cultures in WERI Rb1 compared to scrambled shRNA or parental cells. b,c. The percentage of Ki67-positive cells was reduced by an average of 36% in Y79 (**b**) and 30% in WERI Rb1 (**c**) cells transduced with Nodal shRNAs, compared to scrambled shRNA. P values were calculated using two-sided Student t-test comparing cells expressing Nodal shRNA and scrambled shRNA. Data are presented as mean + SD

Nodal downregulation reduced growth by more than 90%, as determined by CCK-8 growth assay, after 5 days in culture both in Y79 (*p* = 0.001) and in WERI Rb1 cells (*p* < 0.0001) vs scrambled shRNA (Fig. 3a). WERI Rb1 cells transduced with shNodal do not form aggregates or colonies, but they maintain a single cell pattern of growth, while Y79 cells in which Nodal is suppressed form much smaller colonies compared to shScramble or parental cells. Suppression of Nodal also correlated with significant reductions in proliferation as determined by Ki67 immunoassay, with decreases of 36% in Y79 (*p* = 0.02) and 30% in WERI Rb1 cells (*p* < 0.0001) compared to scrambled shRNA controls (Fig. 3b,c).

### Reduction of Nodal inhibits dissemination of Y79 cells in zebrafish

**Fig. 4:**
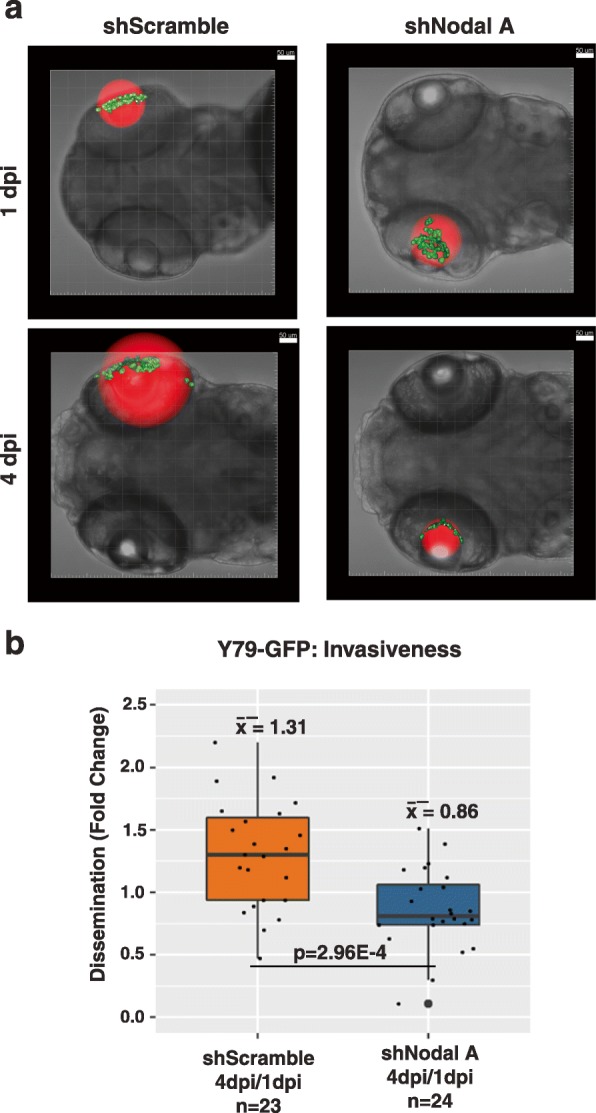
Reduction of Nodal inhibits invasion of Y79 cells in zebrafish. **a** Representative images of the localization of Y79-GFP cells (highlighted in green) expressing Nodal shRNA (sequence A) or scrambled shRNA at 1 and 4 dpi after intravitreal injection in zebrafish. **b** We observed 34% reduction in the fold-change of the minimum bounding sphere (MBS, highlighted in red, 50 μm grid for scale) diameter (μm) between day 1 and day 4 post-injection images when zebrafish larvae (*n* = 24) were injected intravitreally with Y79-GFP cells expressing Nodal shRNA, compared to scrambled shRNA (*n* = 23). Effect size: -0.44; 95% CI: − 0.67, − 0.22; *p* = 2.96 × 10− 4; 50 μm grid for scale

GFP-labelled Y79 cells expressing Nodal shRNA or the scrambled shRNA control were injected intravitreally into the zebrafish eye at 2 days post-fertilization (dpf) to test the effect of reduced Nodal expression on metastatic potential in vivo. Localization of the Y79-GFP cells was monitored longitudinally by confocal microscopy at 1 and 4 days post-injection (dpi). We did not observe any significant increase in cell number during this time period for either condition; however, we did observe differences in dissemination. Control Y79-GFP cells were found to have spread away from the initial injection site by 4 dpi, both within and outside the eye. Conversely, cells expressing Nodal shRNA did not spread away from the initial injection site, largely remaining within the eye when observed at 4 dpi. Minimum bounding sphere (MBS, highlighted in red in Fig. 4a**,** 50 μm grid for scale) diameter was used to determine the extent of tumor dissemination, per our prior report [3]. This analysis showed a 34% decrease in cell spread at 4 dpi in the group of zebrafish injected with Y79-GFP cells expressing Nodal shRNA (*p* = 0.0003, *n* = 23), compared to those injected with cells expressing scrambled shRNA (*n* = 24), (Fig. 4b). These in vivo data provide further evidence that Nodal plays an important role in promoting dissemination of retinoblastoma cells, and therefore it might represent a novel therapeutic target for advanced disease.

## Discussion

We have recently demonstrated a crucial role for ACVR1C/ALK7, a type I receptor of the TGF-β family ligands, in promoting an aggressive phenotype in retinoblastoma [3]. Here we focused on the role of an ACVR1C/ALK7 ligand, Nodal, to determine whether modulation of expression might impact downstream signaling as well as metastatic behavior of the retinoblastoma cells. We specifically focused on Nodal as we previously observed upregulated expression of this ligand at the mRNA and protein levels in multiple retinoblastoma lines, as compared to the other two ligands, Activin and GDF3, which were expressed at lower levels and only in a few of the cell lines tested [3]. We also observed moderate to strong expression of Nodal in about 75% of the primary retinoblastoma samples that we have analyzed by IHC.

Nodal expression is largely restricted to embryonic tissues and is absent from adult non-neoplastic tissues [32]. However, aberrant re-expression of Nodal has a prominent role in tumorigenesis and metastasis in melanoma, glioma, breast, prostate, and pancreatic cancers, with expression levels being directly proportional to tumor grade [16, 18, 19, 32]. Interestingly, a recent report shows that suppression of Nodal significantly reduced growth, clonogenicity, migration and invasion in bladder cancer cells [20]. Surprisingly, when we stimulated WERI Rb1 and Y79 cells with exogenous Nodal at 100, 300, 500 ng/mL, we did not observe any further increase in SMAD2 phosphorylation [3]. Similarly, invasion, proliferation and cell growth were not significantly altered by treatment with exogenous Nodal in these cell lines. Thus, the elevated expression of Nodal present in WERI Rb1 and Y79 cells at steady state may already maximally activate downstream signaling as well as tumorigenesis. To test this hypothesis, we inhibited the endogenous expression of Nodal in WERI Rb1 and Y79 cells, using the shRNA technology, and found more than 90% reduction in the growth rate, a 30 to 36% reduction in proliferation, and from 50 to 80% reduction in transwell invasion. Importantly, analysis in an orthotopic model of retinoblastoma invasion in zebrafish confirmed the role of Nodal in promoting metastatic potential in retinoblastoma, as we observed a significant reduction in tumor spread when the expression of Nodal was suppressed in the injected tumor cells. To increase experimental rigor, we performed our analysis using five different shRNA constructs targeting Nodal mRNA. In both cell lines, all shRNAs were effective in suppressing Nodal expression by more than 90% at the protein level, as well as in abrogating SMAD2 phosphorylation/activation, and in dramatically reducing the protein levels of ZEB1 and Snail, two EMT transcription factors known to play an important role in promoting the invasive properties of the cancer cells [26]. These data are consistent with our previous observation that reduction in invasion, upon pharmacological and genetic inhibition of the ACVR1C receptor, correlated with decrease in ZEB1 and Snail protein levels, in multiple retinoblastoma lines [3]. Other studies have shown that Nodal controls migration and invasion in several tumor types by modulating the expression of Snail, Slug and ZEB1 [8, 10, 12, 36]. We therefore postulate that ZEB1 and Snail might mediate, at least in part, the effects of Nodal signaling on invasion, as we observed that knock down of Nodal, besides reducing ZEB1 and Snail, significantly inhibited metastatic potential both in vitro and in vivo.

Interestingly, we found that Nodal plays an important role in maintaining retinoblastoma cell survival, as we observed a significant induction in the apoptotic markers, such as cleaved PARP1 and in cleaved caspase-3 (using immunoblotting and immunofluorescence assays, respectively), in retinoblastoma cells expressing Nodal shRNA compared to scrambled shRNA.

Potent morphogens, such as Nodal, require tight regulation of their expression and activity to properly control induction of signaling to only those regions where it is needed during embryogenesis. Endogenous antagonists of Nodal, such as the secreted proteins Cerberus and Lefty, play an important role in negatively regulating Nodal activity by preventing the binding of Nodal with its receptors and suppressing Nodal-mediated phenotypes [4, 32]. Therefore, these developmental antagonists of Nodal could represent potential anti-Nodal therapeutics [4, 15]. In addition, Nodal function-blocking antibodies have been shown to be promising tools to inhibit Nodal activity, both in vitro and in a murine model of cutaneous melanoma, where they produced a remarkable reduction in the ability of metastatic melanoma cells to colonize lungs in mice [32]. More recently, a novel monoclonal antibody (3D1), raised against human Nodal, have been shown to inhibit clonogenicity, vasculogenic network formation and human melanoma xenograft growth in immunocompromised mice [33]. Further investigation is therefore warranted to determine the efficacy of these approaches in ameliorating the effects of current therapies in retinoblastoma.

## Conclusions

From these data, we can conclude that Nodal ligand promotes not only invasion but also proliferation, growth, and survival of retinoblastoma cells, and therefore it can be considered a new strategic target to inhibit both primary retinoblastoma growth and extraocular metastatic dissemination.

## Data Availability

Primary research data are presented in the manuscript. No publicly available datasets have been generated as part of this work.
